# Ethyl 4-anilino-2,6-bis­(4-fluoro­phen­yl)-1-phenyl-1,2,5,6-tetra­hydro­pyridine-3-carboxyl­ate

**DOI:** 10.1107/S1600536813002158

**Published:** 2013-01-26

**Authors:** Sumati Anthal, Goutam Brahmachari, Suvankar Das, Rajni Kant, Vivek K. Gupta

**Affiliations:** a Post-Graduate Department of Physics & Electronics, University of Jammu, Jammu Tawi 180 006, India; b Laboratory of Natural Products & Organic Synthesis, Department of Chemistry, Visva-Bharati University, Santiniketan 731 235, West Bengal, India

## Abstract

In the title compound, C_32_H_28_F_2_N_2_O_2_, the tetra­hydro­pyridine ring adopts a distorted boat conformation. The two fluoro­phenyl groups are attached to the tetra­hydro­pyridine ring in a *trans* orientation. The dihedral angle between the planes of the fluoro-substituted rings is 57.0 (1)°. The amino group and carbonyl O atom are involved in intra­molecular hydrogen bonding. In the crystal, weak C—H⋯O, C—H⋯F and C—H⋯π inter­actions link the mol­ecules into columns along [010].

## Related literature

For the crystal structures of related densely functionalized piperidine derivatives, see: Sambyal *et al.* (2011 ▶); Brahmachari & Das (2012 ▶); Khan *et al.* (2008 ▶, 2010 ▶). For general background to functionalized piperidines, see: Desai *et al.* (1992 ▶). For applications of functionalized piperidines, see: Jaen *et al.* (1988 ▶); Schotte *et al.* (1996 ▶); Agrawal & Somani (2009 ▶). For bond-length data in organic compounds, see: Allen *et al.* (1987 ▶).
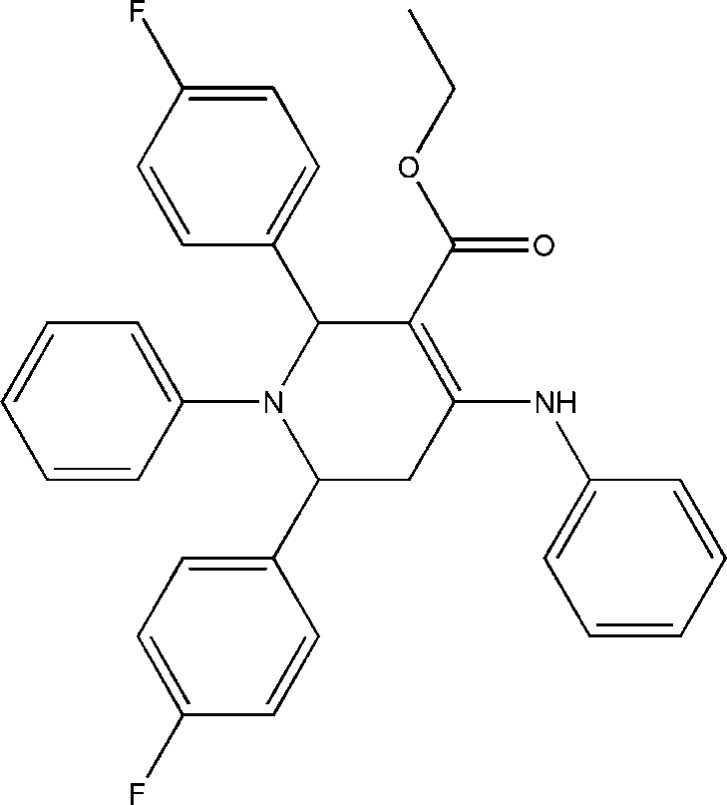


## Experimental

### 

#### Crystal data

C_32_H_28_F_2_N_2_O_2_
*M* *_r_* = 510.56
Triclinic, 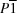

*a* = 10.0432 (4) Å
*b* = 10.4646 (4) Å
*c* = 13.9932 (6) Å
α = 105.422 (4)°
β = 105.982 (4)°
γ = 96.407 (4)°
*V* = 1335.53 (9) Å^3^
*Z* = 2
Mo *K*α radiation
μ = 0.09 mm^−1^
*T* = 293 K
0.30 × 0.20 × 0.20 mm

#### Data collection

Oxford Diffraction Xcalibur Sapphire3 diffractometer
Absorption correction: multi-scan (*CrysAlis PRO*; Oxford Diffraction, 2010 ▶) *T*_min_ = 0.846, *T*_max_ = 1.000
19971 measured reflections
5521 independent reflections
3372 reflections with *I* > 2σ(*I*)
*R*_int_ = 0.041

#### Refinement

*R*[*F* ^2^ > 2σ(*F* ^2^)] = 0.056
*wR*(*F* ^2^) = 0.167
*S* = 1.03
5521 reflections
344 parameters
H-atom parameters constrained
Δρ_max_ = 0.20 e Å^−3^
Δρ_min_ = −0.24 e Å^−3^

### 

Data collection: *CrysAlis PRO* (Oxford Diffraction, 2010 ▶); cell refinement: *CrysAlis PRO*; data reduction: *CrysAlis PRO*; program(s) used to solve structure: *SHELXS97* (Sheldrick, 2008 ▶); program(s) used to refine structure: *SHELXL97* (Sheldrick, 2008 ▶); molecular graphics: *ORTEP-3* (Farrugia, 2012 ▶); software used to prepare material for publication: *PLATON* (Spek, 2009 ▶).

## Supplementary Material

Crystal structure: contains datablock(s) I, global. DOI: 10.1107/S1600536813002158/cv5374sup1.cif

Structure factors: contains datablock(s) I. DOI: 10.1107/S1600536813002158/cv5374Isup2.hkl

Supplementary material file. DOI: 10.1107/S1600536813002158/cv5374Isup3.cml

Additional supplementary materials:  crystallographic information; 3D view; checkCIF report

## Figures and Tables

**Table 1:** Hydrogen-bond geometry (Å, °) *Cg*1 is the centroid of the C10–C15 ring.

*D*—H⋯*A*	*D*—H	H⋯*A*	*D*⋯*A*	*D*—H⋯*A*
N2—H2⋯O1	0.86	2.01	2.672 (3)	133
C11—H11⋯O1^i^	0.93	2.46	3.298 (3)	150
C9—H9*A*⋯F1^ii^	0.96	2.55	3.412 (3)	148
C26—H26⋯*Cg*1^ii^	0.93	2.66	3.470 (3)	146

Symmetry codes: (i) 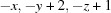; (ii) 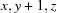.

**Fig. 1.**
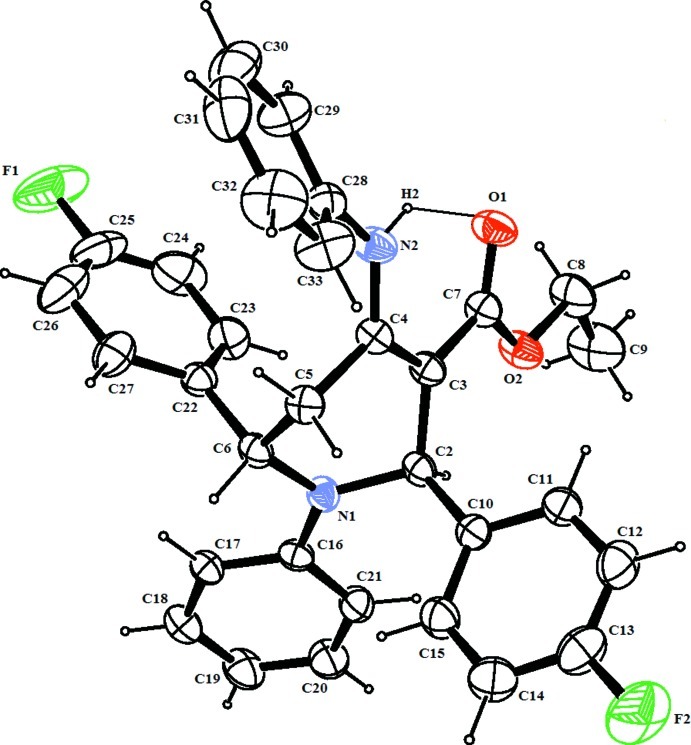
ORTEP view of the molecule with the atom-labeling scheme. The displacement ellipsoids are drawn at the 40% probability level. H atoms are shown as small spheres of arbitrary radii.

## supplementary crystallographic information

### Comment

Functionalized piperidines are found to constitute a very important core in numerous natural products (Desai *et al.*, 1992). In particular, 1,4-disubstituted piperidine scaffolds find useful applications as established drugs (Schotte *et al.*, 1996) and they exhibit a wide range of pharmacological activities including antibacterial, antimalarial, anti-hypertensive, anticonvulsant, anti-inflammatory, and enzyme inhibitory activity (Agrawal & Somani, 2009; Jaen *et al.*, 1988). In continuation of our structural studies of densely functionalized piperidines (Sambyal *et al.*, 2011; Brahmachari & Das, 2012) we present here the title compound, (I).

In (I) (Fig. 1), all bond lengths are within normal ranges (Allen *et al.*, 1987) and comparable to those observed in related structures (Khan *et al.*, 2008, 2010). The dihedral angle between fluoro-substituted phenyl rings are 57.0 (1) °. In the title molecule, tetrahydropyridine ring adopts a distorted bath conformation. The length of the double bond C7=O1 is confirmed by the respective distance of 1.222 (2) Å. The length of the double bond C7=O1 is larger than the standard value for carbonyl group (1.192 Å) and lengthening of the C7=O1 double bond is due to strong intramolecular hydrogen bond between N2 and O1. This intramolecular interaction leads to the formation of a pseudo-six membered ring comprising atoms O1, C7, C3, C4, N2 and H2.

In the crystal, weak intermolecular C—H···O, C—H···F and C—H···π interactions (Table 1) link the molecules into columns in [010].

### Experimental

An oven-dried screw cap reaction tube was charged with a magnetic stir bar, aniline (2 mmol), ethyl acetoacetate (1 mmol) and Bi(NO_3_)_3_.5H_2_O (10 mol%) in 4 ml ethanol; the mixture was stirred at room temperature for 20 min, and after then 4-fluorobenzaldehyde (2 mmol) was added to the reaction mixture and stirring was continued up to 17 h to complete the reaction (monitored by TLC). On completion of the reaction, a thick white precipitate was obtained. The solid residue was filtered off and washed with cold ethanol–water. The solid mass was dissolved in hot ethyl acetate–ethanol mixture and filtered off when bismuth salt separated out; the filtrate on standing afforded white crystals of the title compound, characterized by elemental analyses and spectral studies including FT–IR, ^1^*H*-NMR, and ^13^C-NMR. For X-ray study, single crystals of the title compound were prepared by further recrystallization by slow evaporation from ethanol-ethyl acetate-water solution. White crystals; mp 204–208 °C. ^1^H NMR (400 MHz, CDCl_3_): δH 1.45 (t, 3H, J = 7.2 Hz), 2.73–2.86 (m, 2H), 4.28–4.36 (m, 1H), 4.41–4.49 (m, 1H), 5.11 (d, 1H, J = 2.8 Hz), 6.39 (d, 3H, J = 6.4 Hz), 6.48 (d, 2H, J = 8.4 Hz), 6.63 (t, 1H, J = 7.2 Hz), 6.93- 6.98 (m, 4H), 7.06–7.16 (m, 7H), 7.24–7.29 (m, 2H), 10.31 (s, 1H). ^13^C NMR (100 MHz, CDCl_3_): δC. 14.81, 33.81, 54.61, 57.35, 59.83, 98.05, 113.01, 114.92, 115.14, 115.37, 115.59, 116.58, 125.66, 125.88, 127.87, 127.96, 128.09, 128.17, 128.98, 129.02, 137.76, 138.09, 138.12, 139.48, 139.51, 146.64, 155.91, 160.30, 160.77, 162.74, 163.20, 168.08. IR ν max (KBr): 3234, 3059, 2976, 2924, 2866, 1649, 1591, 1498, 1408, 1259, 1070, 1020, 821, 694, 620 cm^-1^. Anal. Calcd for C_32_ H_28_F_2_N_2_ O_2_: C 75.28, H 5.53, N 5.49; found: C 75.29, H 5.54, N 5.51.

### Refinement

All H atoms were positioned geometrically and were treated as riding on their parent C/N atoms, with C—H distances of 0.93–0.98 Å and N—H distance of 0.86 Å; and with *U*_iso_(H) = 1.2*U*_eq_(C), except for the methyl groups where *U*_iso_(H) = 1.5*U*_eq_(C). There are 14 reflections below theta minimum set for data collection. Some of them have theta less than 2.5 ° which will anyway be blocked collimator. However, some of them could be collected by lowering the minimum theta. The reported value of theta minimum was set by the automatic data collection strategy of the diffractometer software.

### Crystal data

**Table:**

C_32_H_28_F_2_N_2_O_2_	*Z* = 2
*M**_r_* = 510.56	*F*(000) = 536
Triclinic, *P*1	*D*_x_ = 1.270 Mg m^−^^3^
Hall symbol: -P 1	Mo *K*α radiation, λ = 0.71073 Å
*a* = 10.0432 (4) Å	Cell parameters from 6806 reflections
*b* = 10.4646 (4) Å	θ = 3.5–29.1°
*c* = 13.9932 (6) Å	µ = 0.09 mm^−^^1^
α = 105.422 (4)°	*T* = 293 K
β = 105.982 (4)°	Block, white
γ = 96.407 (4)°	0.30 × 0.20 × 0.20 mm
*V* = 1335.53 (9) Å^3^	

### Data collection

**Table:**

Oxford Diffraction Xcalibur Sapphire3 diffractometer	5521 independent reflections
Radiation source: fine-focus sealed tube	3372 reflections with *I* > 2σ(*I*)
Graphite monochromator	*R*_int_ = 0.041
Detector resolution: 16.1049 pixels mm^-1^	θ_max_ = 26.5°, θ_min_ = 3.5°
ω scans	*h* = −12→12
Absorption correction: multi-scan (*CrysAlis PRO*; Oxford Diffraction, 2010)	*k* = −13→13
*T*_min_ = 0.846, *T*_max_ = 1.000	*l* = −17→17
19971 measured reflections	

### Refinement

**Table:**

Refinement on *F*^2^	Primary atom site location: structure-invariant direct methods
Least-squares matrix: full	Secondary atom site location: difference Fourier map
*R*[*F*^2^ > 2σ(*F*^2^)] = 0.056	Hydrogen site location: inferred from neighbouring sites
*wR*(*F*^2^) = 0.167	H-atom parameters constrained
*S* = 1.03	*w* = 1/[σ^2^(*F*_o_^2^) + (0.0731*P*)^2^ + 0.1544*P*] where *P* = (*F*_o_^2^ + 2*F*_c_^2^)/3
5521 reflections	(Δ/σ)_max_ = 0.001
344 parameters	Δρ_max_ = 0.20 e Å^−^^3^
0 restraints	Δρ_min_ = −0.24 e Å^−^^3^

### Special details

**Table:**

Experimental. *CrysAlis PRO*, Oxford Diffraction Ltd., Version 1.171.34.40 (release 27–08-2010 CrysAlis171. NET) (compiled Aug 27 2010,11:50:40) Empirical absorption correction using spherical harmonics, implemented in SCALE3 ABSPACK scaling algorithm.
Geometry. All e.s.d.'s (except the e.s.d. in the dihedral angle between two l.s. planes) are estimated using the full covariance matrix. The cell e.s.d.'s are taken into account individually in the estimation of e.s.d.'s in distances, angles and torsion angles; correlations between e.s.d.'s in cell parameters are only used when they are defined by crystal symmetry. An approximate (isotropic) treatment of cell e.s.d.'s is used for estimating e.s.d.'s involving l.s. planes.
Refinement. Refinement of *F*^2^ against ALL reflections. The weighted *R*-factor *wR* and goodness of fit *S* are based on *F*^2^, conventional *R*-factors *R* are based on *F*, with *F* set to zero for negative *F*^2^. The threshold expression of *F*^2^ > σ(*F*^2^) is used only for calculating *R*-factors(gt) *etc*. and is not relevant to the choice of reflections for refinement. *R*-factors based on *F*^2^ are statistically about twice as large as those based on *F*, and *R*- factors based on ALL data will be even larger.

### Fractional atomic coordinates and isotropic or equivalent isotropic displacement parameters (Å^2^)

**Table:**

	*x*	*y*	*z*	*U*_iso_*/*U*_eq_	
O1	0.06508 (17)	0.83928 (15)	0.48277 (12)	0.0642 (4)	
O2	0.24814 (15)	1.00928 (15)	0.52566 (11)	0.0602 (4)	
N1	0.42208 (16)	0.98911 (15)	0.80637 (13)	0.0435 (4)	
N2	0.02371 (17)	0.75242 (17)	0.63745 (14)	0.0541 (5)	
H2	0.0017	0.7384	0.5711	0.065*	
F1	0.4324 (2)	0.36262 (17)	0.6220 (2)	0.1577 (10)	
F2	0.1378 (2)	1.48695 (18)	0.96125 (17)	0.1255 (7)	
C2	0.32539 (19)	1.04907 (18)	0.73928 (15)	0.0413 (5)	
H2A	0.3781	1.0853	0.6997	0.050*	
C3	0.20278 (19)	0.94002 (19)	0.66044 (15)	0.0426 (5)	
C4	0.13514 (19)	0.85367 (19)	0.69787 (15)	0.0427 (5)	
C5	0.2015 (2)	0.8679 (2)	0.81070 (16)	0.0463 (5)	
H5A	0.1605	0.7911	0.8268	0.056*	
H5B	0.1844	0.9495	0.8542	0.056*	
C6	0.36178 (19)	0.87535 (18)	0.83276 (15)	0.0425 (5)	
H6	0.4050	0.8918	0.9079	0.051*	
C7	0.1637 (2)	0.9234 (2)	0.55011 (17)	0.0475 (5)	
C8	0.2171 (3)	0.9964 (3)	0.41545 (19)	0.0757 (7)	
H8A	0.1266	1.0206	0.3893	0.091*	
H8B	0.2123	0.9037	0.3757	0.091*	
C9	0.3289 (4)	1.0865 (3)	0.4038 (2)	0.1045 (11)	
H9A	0.3346	1.1777	0.4450	0.157*	
H9B	0.3083	1.0816	0.3316	0.157*	
H9C	0.4174	1.0597	0.4273	0.157*	
C10	0.2753 (2)	1.16718 (18)	0.80160 (16)	0.0430 (5)	
C11	0.1627 (2)	1.2155 (2)	0.75133 (19)	0.0563 (6)	
H11	0.1175	1.1747	0.6800	0.068*	
C12	0.1156 (3)	1.3227 (2)	0.8043 (2)	0.0719 (7)	
H12	0.0397	1.3544	0.7697	0.086*	
C13	0.1829 (3)	1.3806 (2)	0.9080 (2)	0.0740 (7)	
C14	0.2956 (3)	1.3388 (3)	0.9614 (2)	0.0745 (7)	
H14	0.3408	1.3815	1.0325	0.089*	
C15	0.3412 (3)	1.2309 (2)	0.90674 (18)	0.0585 (6)	
H15	0.4180	1.2008	0.9419	0.070*	
C16	0.56767 (19)	1.03852 (19)	0.84332 (15)	0.0420 (5)	
C17	0.6603 (2)	0.9685 (2)	0.89330 (15)	0.0447 (5)	
H17	0.6241	0.8888	0.9028	0.054*	
C18	0.8046 (2)	1.0161 (2)	0.92869 (18)	0.0570 (6)	
H18	0.8642	0.9680	0.9618	0.068*	
C19	0.8616 (2)	1.1337 (2)	0.9157 (2)	0.0693 (7)	
H19	0.9589	1.1648	0.9388	0.083*	
C20	0.7717 (2)	1.2039 (2)	0.8678 (2)	0.0671 (7)	
H20	0.8089	1.2840	0.8592	0.081*	
C21	0.6271 (2)	1.1582 (2)	0.83221 (17)	0.0536 (6)	
H21	0.5686	1.2081	0.8003	0.064*	
C22	0.3867 (2)	0.73939 (19)	0.77545 (16)	0.0451 (5)	
C23	0.4137 (3)	0.7159 (2)	0.68185 (19)	0.0653 (6)	
H23	0.4226	0.7862	0.6536	0.078*	
C24	0.4279 (3)	0.5881 (3)	0.6291 (2)	0.0934 (10)	
H24	0.4452	0.5716	0.5654	0.112*	
C25	0.4157 (3)	0.4868 (3)	0.6732 (3)	0.0967 (11)	
C26	0.3936 (3)	0.5079 (3)	0.7662 (3)	0.0910 (10)	
H26	0.3896	0.4383	0.7958	0.109*	
C27	0.3772 (2)	0.6339 (2)	0.8168 (2)	0.0667 (7)	
H27	0.3592	0.6486	0.8802	0.080*	
C28	−0.0612 (2)	0.6666 (2)	0.67033 (18)	0.0496 (5)	
C29	−0.1048 (3)	0.5324 (2)	0.6143 (2)	0.0746 (7)	
H29	−0.0776	0.4974	0.5558	0.089*	
C30	−0.1896 (3)	0.4487 (3)	0.6452 (3)	0.0919 (10)	
H30	−0.2192	0.3574	0.6069	0.110*	
C31	−0.2302 (3)	0.4971 (4)	0.7296 (3)	0.0927 (10)	
H31	−0.2850	0.4396	0.7509	0.111*	
C32	−0.1896 (3)	0.6314 (4)	0.7833 (3)	0.0997 (10)	
H32	−0.2187	0.6660	0.8409	0.120*	
C33	−0.1066 (3)	0.7165 (3)	0.7540 (2)	0.0779 (8)	
H33	−0.0810	0.8084	0.7910	0.094*	

### Atomic displacement parameters (Å^2^)

**Table:**

	*U*^11^	*U*^22^	*U*^33^	*U*^12^	*U*^13^	*U*^23^
O1	0.0674 (10)	0.0715 (10)	0.0401 (9)	−0.0066 (8)	0.0064 (8)	0.0150 (7)
O2	0.0605 (9)	0.0788 (10)	0.0398 (9)	−0.0019 (8)	0.0154 (7)	0.0229 (7)
N1	0.0408 (9)	0.0471 (9)	0.0449 (10)	0.0064 (7)	0.0120 (7)	0.0207 (8)
N2	0.0500 (10)	0.0623 (11)	0.0438 (11)	−0.0046 (8)	0.0126 (8)	0.0151 (8)
F1	0.1198 (16)	0.0709 (11)	0.224 (3)	0.0209 (10)	0.0456 (16)	−0.0400 (13)
F2	0.1365 (16)	0.1095 (13)	0.1386 (18)	0.0639 (12)	0.0714 (14)	0.0069 (12)
C2	0.0420 (10)	0.0494 (11)	0.0349 (11)	0.0056 (8)	0.0127 (8)	0.0182 (9)
C3	0.0412 (11)	0.0508 (11)	0.0357 (11)	0.0061 (8)	0.0129 (9)	0.0138 (9)
C4	0.0396 (10)	0.0502 (11)	0.0398 (12)	0.0089 (9)	0.0153 (9)	0.0136 (9)
C5	0.0481 (12)	0.0515 (11)	0.0440 (12)	0.0077 (9)	0.0209 (9)	0.0168 (9)
C6	0.0456 (11)	0.0506 (11)	0.0329 (11)	0.0061 (9)	0.0124 (9)	0.0169 (9)
C7	0.0475 (12)	0.0522 (12)	0.0444 (13)	0.0081 (9)	0.0166 (10)	0.0161 (10)
C8	0.0909 (19)	0.0969 (19)	0.0430 (15)	0.0081 (15)	0.0225 (13)	0.0308 (13)
C9	0.121 (3)	0.130 (3)	0.066 (2)	−0.007 (2)	0.0425 (19)	0.0371 (18)
C10	0.0433 (11)	0.0439 (10)	0.0437 (12)	0.0040 (8)	0.0148 (9)	0.0180 (9)
C11	0.0446 (12)	0.0547 (13)	0.0625 (15)	0.0062 (10)	0.0086 (11)	0.0171 (11)
C12	0.0525 (14)	0.0662 (15)	0.098 (2)	0.0196 (12)	0.0213 (14)	0.0261 (15)
C13	0.0787 (18)	0.0629 (15)	0.090 (2)	0.0252 (14)	0.0453 (17)	0.0149 (15)
C14	0.095 (2)	0.0770 (17)	0.0530 (16)	0.0256 (15)	0.0316 (14)	0.0117 (13)
C15	0.0678 (15)	0.0645 (14)	0.0468 (14)	0.0206 (11)	0.0191 (11)	0.0192 (11)
C16	0.0423 (11)	0.0475 (11)	0.0338 (11)	0.0052 (8)	0.0128 (9)	0.0096 (8)
C17	0.0455 (11)	0.0519 (11)	0.0372 (11)	0.0092 (9)	0.0124 (9)	0.0159 (9)
C18	0.0459 (12)	0.0739 (15)	0.0501 (14)	0.0136 (11)	0.0108 (10)	0.0214 (11)
C19	0.0428 (13)	0.0845 (17)	0.0729 (18)	−0.0013 (12)	0.0068 (12)	0.0299 (14)
C20	0.0549 (14)	0.0659 (14)	0.0740 (18)	−0.0060 (11)	0.0097 (13)	0.0296 (13)
C21	0.0499 (12)	0.0525 (12)	0.0559 (14)	0.0029 (10)	0.0089 (10)	0.0238 (10)
C22	0.0402 (11)	0.0470 (11)	0.0452 (12)	0.0036 (8)	0.0115 (9)	0.0137 (9)
C23	0.0730 (16)	0.0738 (15)	0.0536 (15)	0.0261 (12)	0.0255 (12)	0.0170 (12)
C24	0.086 (2)	0.107 (2)	0.068 (2)	0.0378 (18)	0.0240 (16)	−0.0102 (17)
C25	0.0681 (18)	0.0488 (16)	0.136 (3)	0.0092 (13)	0.0179 (19)	−0.0149 (18)
C26	0.0741 (19)	0.0514 (16)	0.148 (3)	0.0066 (13)	0.043 (2)	0.0268 (18)
C27	0.0667 (15)	0.0553 (14)	0.0875 (19)	0.0092 (11)	0.0341 (14)	0.0288 (13)
C28	0.0377 (11)	0.0551 (13)	0.0563 (14)	0.0060 (9)	0.0136 (10)	0.0207 (10)
C29	0.0713 (17)	0.0625 (15)	0.085 (2)	0.0021 (12)	0.0329 (15)	0.0119 (14)
C30	0.0710 (18)	0.0629 (17)	0.140 (3)	0.0017 (14)	0.030 (2)	0.0377 (18)
C31	0.0594 (17)	0.113 (3)	0.136 (3)	0.0133 (17)	0.0367 (19)	0.084 (2)
C32	0.083 (2)	0.120 (3)	0.112 (3)	−0.0001 (18)	0.0578 (19)	0.041 (2)
C33	0.0705 (16)	0.0800 (17)	0.089 (2)	0.0029 (13)	0.0479 (15)	0.0169 (15)

### Geometric parameters (Å, º)

**Table:**

O1—C7	1.222 (2)	C14—C15	1.386 (3)
O2—C7	1.341 (2)	C14—H14	0.9300
O2—C8	1.452 (3)	C15—H15	0.9300
N1—C16	1.395 (2)	C16—C21	1.392 (3)
N1—C6	1.455 (2)	C16—C17	1.399 (3)
N1—C2	1.471 (2)	C17—C18	1.381 (3)
N2—C4	1.352 (2)	C17—H17	0.9300
N2—C28	1.418 (3)	C18—C19	1.376 (3)
N2—H2	0.8600	C18—H18	0.9300
F1—C25	1.360 (3)	C19—C20	1.371 (3)
F2—C13	1.362 (3)	C19—H19	0.9300
C2—C3	1.518 (3)	C20—C21	1.380 (3)
C2—C10	1.532 (3)	C20—H20	0.9300
C2—H2A	0.9800	C21—H21	0.9300
C3—C4	1.365 (3)	C22—C23	1.374 (3)
C3—C7	1.441 (3)	C22—C27	1.382 (3)
C4—C5	1.494 (3)	C23—C24	1.389 (4)
C5—C6	1.543 (3)	C23—H23	0.9300
C5—H5A	0.9700	C24—C25	1.371 (5)
C5—H5B	0.9700	C24—H24	0.9300
C6—C22	1.521 (3)	C25—C26	1.345 (5)
C6—H6	0.9800	C26—C27	1.373 (4)
C8—C9	1.457 (4)	C26—H26	0.9300
C8—H8A	0.9700	C27—H27	0.9300
C8—H8B	0.9700	C28—C29	1.367 (3)
C9—H9A	0.9600	C28—C33	1.368 (3)
C9—H9B	0.9600	C29—C30	1.385 (4)
C9—H9C	0.9600	C29—H29	0.9300
C10—C15	1.375 (3)	C30—C31	1.345 (5)
C10—C11	1.381 (3)	C30—H30	0.9300
C11—C12	1.379 (3)	C31—C32	1.359 (4)
C11—H11	0.9300	C31—H31	0.9300
C12—C13	1.353 (4)	C32—C33	1.369 (4)
C12—H12	0.9300	C32—H32	0.9300
C13—C14	1.360 (4)	C33—H33	0.9300
			
C7—O2—C8	116.64 (17)	C13—C14—H14	121.0
C16—N1—C6	120.52 (15)	C15—C14—H14	121.0
C16—N1—C2	121.37 (15)	C10—C15—C14	121.5 (2)
C6—N1—C2	118.11 (15)	C10—C15—H15	119.3
C4—N2—C28	127.81 (18)	C14—C15—H15	119.3
C4—N2—H2	116.1	C21—C16—N1	121.98 (17)
C28—N2—H2	116.1	C21—C16—C17	117.20 (18)
N1—C2—C3	110.00 (14)	N1—C16—C17	120.82 (17)
N1—C2—C10	112.99 (15)	C18—C17—C16	121.00 (19)
C3—C2—C10	112.16 (15)	C18—C17—H17	119.5
N1—C2—H2A	107.1	C16—C17—H17	119.5
C3—C2—H2A	107.1	C19—C18—C17	121.0 (2)
C10—C2—H2A	107.1	C19—C18—H18	119.5
C4—C3—C7	121.12 (18)	C17—C18—H18	119.5
C4—C3—C2	117.10 (18)	C20—C19—C18	118.5 (2)
C7—C3—C2	121.69 (17)	C20—C19—H19	120.7
N2—C4—C3	123.90 (19)	C18—C19—H19	120.7
N2—C4—C5	120.37 (17)	C19—C20—C21	121.4 (2)
C3—C4—C5	115.44 (17)	C19—C20—H20	119.3
C4—C5—C6	108.66 (16)	C21—C20—H20	119.3
C4—C5—H5A	110.0	C20—C21—C16	120.9 (2)
C6—C5—H5A	110.0	C20—C21—H21	119.5
C4—C5—H5B	110.0	C16—C21—H21	119.5
C6—C5—H5B	110.0	C23—C22—C27	118.5 (2)
H5A—C5—H5B	108.3	C23—C22—C6	122.42 (19)
N1—C6—C22	114.26 (16)	C27—C22—C6	119.0 (2)
N1—C6—C5	109.69 (15)	C22—C23—C24	120.6 (3)
C22—C6—C5	109.08 (15)	C22—C23—H23	119.7
N1—C6—H6	107.9	C24—C23—H23	119.7
C22—C6—H6	107.9	C25—C24—C23	118.4 (3)
C5—C6—H6	107.9	C25—C24—H24	120.8
O1—C7—O2	121.4 (2)	C23—C24—H24	120.8
O1—C7—C3	125.04 (19)	C26—C25—F1	119.6 (4)
O2—C7—C3	113.54 (18)	C26—C25—C24	122.3 (3)
O2—C8—C9	108.4 (2)	F1—C25—C24	118.0 (4)
O2—C8—H8A	110.0	C25—C26—C27	118.8 (3)
C9—C8—H8A	110.0	C25—C26—H26	120.6
O2—C8—H8B	110.0	C27—C26—H26	120.6
C9—C8—H8B	110.0	C26—C27—C22	121.3 (3)
H8A—C8—H8B	108.4	C26—C27—H27	119.3
C8—C9—H9A	109.5	C22—C27—H27	119.3
C8—C9—H9B	109.5	C29—C28—C33	119.1 (2)
H9A—C9—H9B	109.5	C29—C28—N2	119.4 (2)
C8—C9—H9C	109.5	C33—C28—N2	121.4 (2)
H9A—C9—H9C	109.5	C28—C29—C30	119.6 (3)
H9B—C9—H9C	109.5	C28—C29—H29	120.2
C15—C10—C11	117.81 (19)	C30—C29—H29	120.2
C15—C10—C2	122.34 (18)	C31—C30—C29	121.2 (3)
C11—C10—C2	119.83 (18)	C31—C30—H30	119.4
C12—C11—C10	121.6 (2)	C29—C30—H30	119.4
C12—C11—H11	119.2	C30—C31—C32	118.8 (3)
C10—C11—H11	119.2	C30—C31—H31	120.6
C13—C12—C11	118.2 (2)	C32—C31—H31	120.6
C13—C12—H12	120.9	C31—C32—C33	121.2 (3)
C11—C12—H12	120.9	C31—C32—H32	119.4
C12—C13—C14	122.8 (2)	C33—C32—H32	119.4
C12—C13—F2	118.9 (3)	C28—C33—C32	120.1 (3)
C14—C13—F2	118.3 (3)	C28—C33—H33	120.0
C13—C14—C15	118.0 (2)	C32—C33—H33	120.0
			
C16—N1—C2—C3	145.42 (17)	C11—C10—C15—C14	0.9 (3)
C6—N1—C2—C3	−34.1 (2)	C2—C10—C15—C14	179.02 (19)
C16—N1—C2—C10	−88.4 (2)	C13—C14—C15—C10	0.1 (4)
C6—N1—C2—C10	92.11 (19)	C6—N1—C16—C21	−170.75 (19)
N1—C2—C3—C4	48.6 (2)	C2—N1—C16—C21	9.8 (3)
C10—C2—C3—C4	−78.1 (2)	C6—N1—C16—C17	9.4 (3)
N1—C2—C3—C7	−128.23 (19)	C2—N1—C16—C17	−170.11 (18)
C10—C2—C3—C7	105.1 (2)	C21—C16—C17—C18	−0.8 (3)
C28—N2—C4—C3	−173.38 (19)	N1—C16—C17—C18	179.09 (18)
C28—N2—C4—C5	13.1 (3)	C16—C17—C18—C19	−0.1 (3)
C7—C3—C4—N2	−3.7 (3)	C17—C18—C19—C20	0.8 (4)
C2—C3—C4—N2	179.45 (17)	C18—C19—C20—C21	−0.7 (4)
C7—C3—C4—C5	170.13 (17)	C19—C20—C21—C16	−0.3 (4)
C2—C3—C4—C5	−6.7 (2)	N1—C16—C21—C20	−178.9 (2)
N2—C4—C5—C6	127.31 (18)	C17—C16—C21—C20	1.0 (3)
C3—C4—C5—C6	−46.8 (2)	N1—C6—C22—C23	−23.4 (3)
C16—N1—C6—C22	−73.6 (2)	C5—C6—C22—C23	99.8 (2)
C2—N1—C6—C22	105.88 (19)	N1—C6—C22—C27	158.97 (18)
C16—N1—C6—C5	163.55 (16)	C5—C6—C22—C27	−77.9 (2)
C2—N1—C6—C5	−16.9 (2)	C27—C22—C23—C24	1.4 (3)
C4—C5—C6—N1	58.4 (2)	C6—C22—C23—C24	−176.3 (2)
C4—C5—C6—C22	−67.4 (2)	C22—C23—C24—C25	−0.7 (4)
C8—O2—C7—O1	−0.4 (3)	C23—C24—C25—C26	−1.4 (5)
C8—O2—C7—C3	178.66 (19)	C23—C24—C25—F1	−178.8 (2)
C4—C3—C7—O1	4.5 (3)	F1—C25—C26—C27	179.9 (2)
C2—C3—C7—O1	−178.82 (19)	C24—C25—C26—C27	2.6 (5)
C4—C3—C7—O2	−174.60 (17)	C25—C26—C27—C22	−1.8 (4)
C2—C3—C7—O2	2.1 (3)	C23—C22—C27—C26	−0.2 (3)
C7—O2—C8—C9	−173.5 (2)	C6—C22—C27—C26	177.6 (2)
N1—C2—C10—C15	15.4 (3)	C4—N2—C28—C29	−140.2 (2)
C3—C2—C10—C15	140.5 (2)	C4—N2—C28—C33	43.1 (3)
N1—C2—C10—C11	−166.45 (17)	C33—C28—C29—C30	−2.2 (4)
C3—C2—C10—C11	−41.4 (2)	N2—C28—C29—C30	−179.0 (2)
C15—C10—C11—C12	−0.9 (3)	C28—C29—C30—C31	−0.1 (4)
C2—C10—C11—C12	−179.12 (19)	C29—C30—C31—C32	1.8 (5)
C10—C11—C12—C13	0.0 (4)	C30—C31—C32—C33	−1.3 (5)
C11—C12—C13—C14	1.0 (4)	C29—C28—C33—C32	2.7 (4)
C11—C12—C13—F2	179.8 (2)	N2—C28—C33—C32	179.4 (3)
C12—C13—C14—C15	−1.1 (4)	C31—C32—C33—C28	−1.0 (5)
F2—C13—C14—C15	−179.8 (2)		

### Hydrogen-bond geometry (Å, º)

Cg1 is the centroid of the C10–C15 ring.

**Table:**

*D*—H···*A*	*D*—H	H···*A*	*D*···*A*	*D*—H···*A*
N2—H2···O1	0.86	2.01	2.672 (3)	133
C11—H11···O1^i^	0.93	2.46	3.298 (3)	150
C9—H9*A*···F1^ii^	0.96	2.55	3.412 (3)	148
C26—H26···*Cg*1^ii^	0.93	2.66	3.470 (3)	146

Symmetry codes: (i) −*x*, −*y*+2, −*z*+1; (ii) *x*, *y*+1, *z*.
